# Deacetyl xylopic acid exhibits synergistic antidepressant-like effects and augments fluoxetine activity in a chronic restraint-induced depression model in mice

**DOI:** 10.1007/s00210-026-05501-8

**Published:** 2026-06-05

**Authors:** Charles Kwaku Benneh, Wonder Kofi Mensah Abotsi, Aliu Moomin, Robert Peter Biney, Japhat Opoku Ofosu, Mustapha Kobina Abeka, Augustine Tandoh, Donatus Wewura Adongo, Eric Woode

**Affiliations:** 1 Department of Clinical Pharmacy and Pharmacy Practice, School of Pharmacy and Pharmaceutical Sciences, Ulster University, Coleraine, UK; 2 Department of Pharmacology and Toxicology, School of Pharmacy and Pharmaceutical Sciences, Kwame Nkrumah University of Science and Technology, Kumasi, Ghana; 3 Rowett Institute, University of Aberdeen, Aberdeen, UK; 4 Department of Pharmacotherapeutics and Pharmacy Practice, School of Pharmacy and Pharmaceutical Sciences, University of Cape Coast, Cape Coast, Ghana; 5 Department of Pharmaceutical Sciences, Faculty of Applied Science and Technology, Sunyani Technical University, Sunyani, Ghana; 6 Department of Pharmaceutical Chemistry, School of Pharmacy and Pharmaceutical Sciences, University of Cape Coast, Cape Coast, Ghana; 7 Graduate School of Pharmaceutical Sciences, Sojo University, Kumamoto, Japan; 8 Department of Pharmacology and Toxicology, School of Pharmacy, University of Health and Allied Sciences, Ho, Ghana

**Keywords:** Xylopic acid, Chronic stress, Isobolograph, Chronic restrain stress

## Abstract

Xylopic acid (XA) and the deacetyl derivative (dXA) have been shown to exhibit antidepressant activity in acute models of depression in mice. This study evaluated and compared the effect of the deacetyl derivative in a chronic restrain model of depression. In the first phase of the study, the synergistic potential of dXA was evaluated using the mouse tail suspension test (TST) and forced swim test (FST). In the second phase, mice were subjected to chronic restraint stress for 42 days, starting from day 0, in ventilated tubes (0800–1400 h). On day 42, stress-naïve and stressed mice were assessed using both the FST and TST to identify the more sensitive behavioural assay for subsequent evaluation of treatment effects. Stressed mice were then administered fluoxetine, XA, dXA, vehicle for 72 h or single acute dosing of combinations of fluoxetine with XA or dXA, followed by repeat behavioural testing 90 min after the last dose of either a single acute or a chronic dosing regimen. The interaction index (*γ*), together with the significantly lower ED_50mix_ compared to the ED_50add_, for the dXA combinations, with fluoxetine, sertraline, imipramine and ketamine is indicative of synergistic effect. An 8-hourly dosing, for 72 h of XA (100 mg kg^−1^) and dXA (100 mg kg^−1^) in the acute models did not reverse the behavioural traits of chronic restrain. However, fluoxetine (30 mg kg^−1^), given 12 hourly for 72 h, and the acute administration of combinations of fluoxetine (100 mg kg^−1^) and dXA (100 mg kg^−1^) significantly reversed the duration of immobility. The present study demonstrates that deacetyl xylopic acid (dXA) exerts synergistic antidepressant—like effects with selective antidepressants and augments the antidepressant effects of fluoxetine in a mouse model of chronic restraint stress-induced depression. These findings suggest that dXA may represent a promising adjunctive agent for improving antidepressant efficacy, particularly in stress-related depressive disorders.

**Supplementary information:**

The online version contains supplementary material available at 10.1007/s00210-026-05501-8.

## Introduction

Drug discovery efforts towards identifying efficacious small lead molecules effective for the management of affective disorders including depression is still relevant due to the significant impact it has at the individual and economic level. Major depressive disorder is one of the most common and burdensome psychiatric disorders that affects more than 300 million individuals worldwide.

Xylopic acid (15β-(acetyloxy)kaure-16-en-18-oic acid) is one of the principal chemical constituents of the ethanolic fruit extract of *Xylopia aethiopica*. Xylopic acid has been shown by several authors to have significant in vivo anti-inflammatory, anxiolytic AND antidepressant effects. The deacetyl derivative was derived from the product of the base catalysis of xylopic acid and was first described by Kofie et al. (2016), as part of a series of synthetic derivatives semi-synthesis with the aim of enhancing antimicrobial activity.

The antidepressant effect has been explored in a previous study where it was established that xylopic acid and the deacetyl derivatives exhibit antidepressant-like effects comparable to fluoxetine, imipramine and sertraline—albeit less potent—mediated, at least partly, through serotoninergic systems.

It was also recently demonstrated by Benneh et al. (2024) the synergistic antidepressant-like effect of xylopic acid when co-administered with selected selective serotonin re-uptake inhibitors (SSRIs) antidepressants (Benneh et al. 2024).

The initial phase of antidepressant-like activity screening relies mainly on the effect of compounds at reducing immobility after confining rodents in an inescapable environment. However, a more robust approach is to demonstrate the reversal of a behavioural deficit after subjecting mice to prolonged stressing routine or injection systemic stress-inducing agents such as lipopolysaccharide (Wang et al. 2020; Lee et al. 2018; Monleon et al. 1995).

Chronic restrain stress, which involves subjecting rodents to restrained movements in an enclosed space for several hours over a number of weeks, produces similar behavioural, biological and biochemical changes that reflect chronic depression (Voorhees et al. 2013). Chronic stress models appear to provide a more realistic model of depression with accompanying behavioural changes, cognitive deficits, molecular alterations and have a higher predictive value compared to acute models (Mineur et al. 2006; Monleon et al. 1995; Voorhees et al. 2013; Willner et al. 1992).

The use of combined antidepressant regimens is increasingly recognised as a strategy to enhance therapeutic efficacy while mitigating adverse effects. Such combinations may confer additive or synergistic benefits through the amplification of shared pharmacological pathways or the complementary engagement of distinct mechanisms of action. It is therefore advantageous to determine early in the discovery process whether this potential exists, as such insight can more effectively guide its evaluation and optimization in later stages of drug development.

This study first evaluated and compared the potential synergistic antidepressant-like effect of dXA to XA in an acute antidepressant-like model in mice. Subsequently, we investigated the potential of XA and dXA reversing the immobility time and weight changes in the chronic restrain stress model. Finally, we investigated the effect of XA or dXA with an SSRI combination in reversing the behavioural symptoms induced by the chronic restrain stress model.

## Materials

### Animals

Male ICR mice (20–30 g) were obtained from the vivarium of the Department of Pharmacology of Kwame Nkrumah University of Science and Technology, Kumasi, Ghana. The housing conditions were as follows: the temperature was 23–25 °C, and the relative humidity was 60–70% in an approximately 12-h light–dark cycle. Animals were allowed free access to a pellet diet and access to water. Approval for this study was obtained from the Research and Ethics Committee with approval number UHAS-REC A.11 [6] 18–19.

### Extraction of xylopic acid (XA) and synthesis of deacetyl xylopic acid (dXA)

**Fig. 1:**
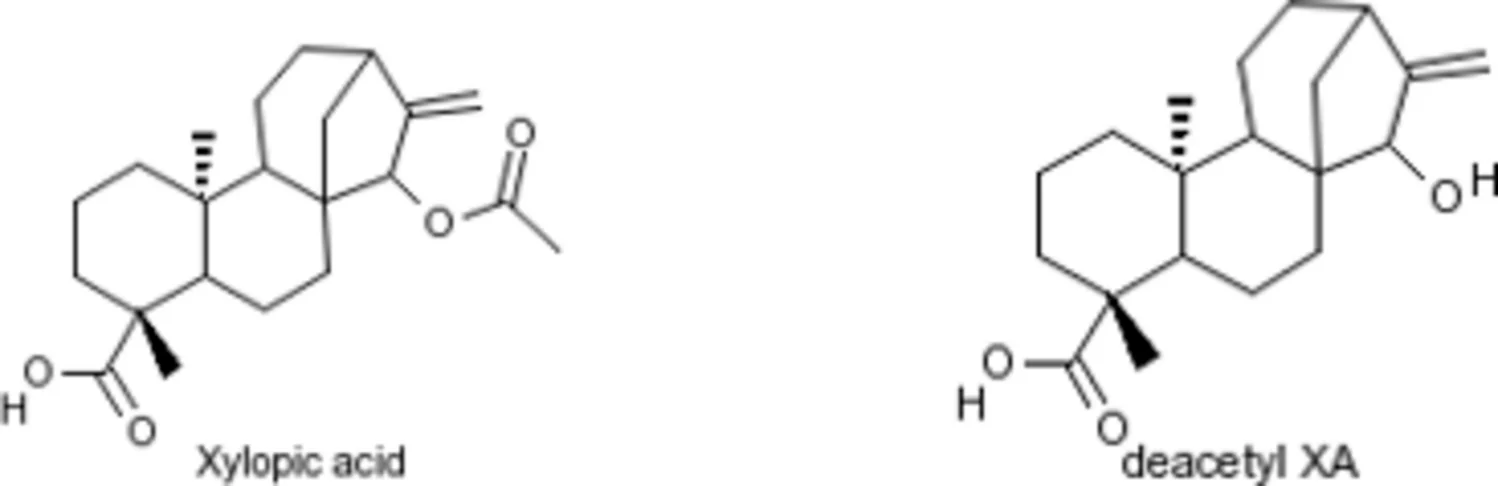
Chemical structure of xylopic acid (15β-(acetyloxy)kaur-16-en-18-oic acid). PubChem CID = 354,614, molar mass = 360.5 gmol^−1^ and the deacetylated derivative

Plant collection, processing and extraction of xylopic were carried according to methods described by Benneh et al. (2025). The deacetyl derivative of xylopic was synthesised and analysed using the adopted methods published earlier (Benneh et al. 2025; Kofie et al. 2016) (Fig. 1).

### Drugs, chemicals and preparation

Ketamine hydrochloride was purchased from Mylan Institutional LLC (Rockford, IL, USA); fluoxetine from Eli Lilly and Co. (Indianapolis, IN, USA); imipramine and sertraline from Accord (Barnstaple, EX32 8NS, UK) and polysorbate 80 from Sigma-Aldrich (St. Louis, MO, USA).

XA, fluoxetine, imipramine and sertraline were prepared by solubilising the fine powder with polysorbate 80 q.s before oral administration. Ketamine was diluted in normal saline before dosing via the intraperitoneal route. A maximum of 0.2 mL was delivered with the aid of an oral gavage for administration *per os* and 0.1 mL for intraperitoneal injections.

## Methods

### Combination study

#### Estimating ED_50_ values for XA, dXA and selected antidepressants

The ED_50_ of dXA and the other compounds of interest were determined in mice following the methods described by Benneh et al. (2025). Mice were dosed with XA (10, 30, 100 mg kg^−1^, *p.o.*), dXA (10, 30, 100 mg kg^−1^, *p.o.*), imipramine (10, 30, 100 mg kg^−1^, *p.o.*), fluoxetine (3, 10, 30 mg kg^−1^, *p.o.*), ketamine (0.1, 0.3, 1.0 mg kg^−1^, *i.p.*), sertraline (3, 10, 30 mg kg^−1^, *p.o.*) or vehicle (10 mL kg^−1^) prior to testing in the forced swim and tail suspension test. Testing was carried out 60 min after administration of fluoxetine, sertraline, imipramine and ketamine or 90 min after XA or dXA dosing.

The total time of immobility was converted to percentage reduction in immobility compared to solvent-treated controlusing theformula:

$$\%\;reduction\;in\;mobility=\frac{IT_c-IT_t}{IT_c}\times\;100\%$$

where:

- IT_c_ = immobility time of control mice
- IT_t_ = immobility time of treated mice

The ED_50_ values of each compound were determined using non-linear regression analysis of the dose–response curves.

#### Isobolographic analysis

The synergistic antidepressant-like effects of XA in combination with fluoxetine, imipramine, sertraline and ketamine have previously been demonstrated by Benneh et al. (2024) using the forced swim test. However, no studies have employed an isobologram approach to assess potential synergism between antidepressants in the tail suspension test. Consequently, the methodology for drug combination and testing in the tail suspension test was adapted from the forced swim protocol described by Benneh et al. (2024), while the execution of the tail suspension test followed the procedures outlined in Benneh et al. (2025).

To further characterise the pharmacological interaction between dXA and these selected antidepressants, an isobolographic analysis was conducted following the procedures outlined by Tallarida (2006), Muller et al. (2015) and Benneh et al. (2024).

Drug interactions were evaluated by administering dXA concurrently with each antidepressant at fixed fractions of their respective ED₅₀ values (0.5:0.5, 0.25:0.25 and 0.125:0.125). The table below presents the ED₅₀ values and the corresponding fractional doses used in the combination experiments. Dose–response curves were generated for each drug mixture, and the ED₅₀ of the combination (ED₅₀_mix) was determined by non-linear regression of the log dose–response relationship. The theoretical additive ED₅₀ (ED₅₀_add) for each mixture was subsequently calculated using the following equation:

$${\mathrm{E}\mathrm{D}}_{50\mathrm{a}\mathrm{d}\mathrm{d}}=f \left(A\right)+\left(1-f\right)B$$

where *A* is the ED_50_ of dXA and *B* is the ED_50_ of each antidepressant alone and *f* denotes the fraction of the ED_50_ in the corresponding mixture.

The variance (Var) of the ED_50_values was calculated using the formula below:

$$VarED_{50}\;add=\;f^2\left(VarA\right)+\left(1-f\right)^2VarB$$

where VarA represents the variance of the ED_50_ values of dXA and VarB is the variance of the ED_50_ values of each antidepressant.

Differences between the theoretical and experimental ED_50_ values were assessed using a Students’ *t-*test. When ED_50add_ is significantly higher than the ED_50mix_, the combination has synergistic interaction.

The magnitude of interaction (*γ*) between the various combinations was calculated using the equation:

$$\gamma=\frac aA+\frac bB$$

where *A* and *B* represent the ED_50_ of each drug when used alone and *a* and *b* are the amounts of each drug in the combination (ED_50mix_).

The interaction is considered additive if *γ* = 1, synergistic if *γ* < 1 and antagonistic if *γ* > 1.

#### Open field test

To exclude the possibility that the tested compound combinations produced nonspecific effects on locomotor activity, mice received an oral administration of either vehicle or the ED₅₀ combinations described in the “Isobolographic analysis” section and were evaluated 90 min later in the open field test. Locomotor activity was quantified as the total distance travelled during a 5‑min session, which served as the primary measure of potential motor effects induced by the drug combinations.

### Chronic restrain stress model of depression

#### Effect of acute and chronic administration

Chronic restraint stress was conducted following the procedures of Voorhees et al. (2013), with specific modifications. The primary alteration involved extending the stress exposure period from 28 to 42 days, accompanied by a shortened post‑stress behavioural assessment window. Additionally, the weekly intermittent forced swim testing described in the original protocol was replaced with a simultaneous behavioural assessment on day 42 using the tail suspension test followed immediately by the forced swim test.

In brief, male ICR mice were randomly allocated to either a control group or a restraint‑stress group after a 7‑day acclimation period. Animals assigned to the restraint‑stress condition were placed individually into well‑ventilated 50-mL Falcon® polypropylene tubes daily from 0800 to 1400 h for 42 consecutive days. At the end of each 6‑h session, both control and stressed mice, having been deprived of food and water during the restraint period, were returned to their home cages and given ad libitum access to food and water.

**Fig. 2:**
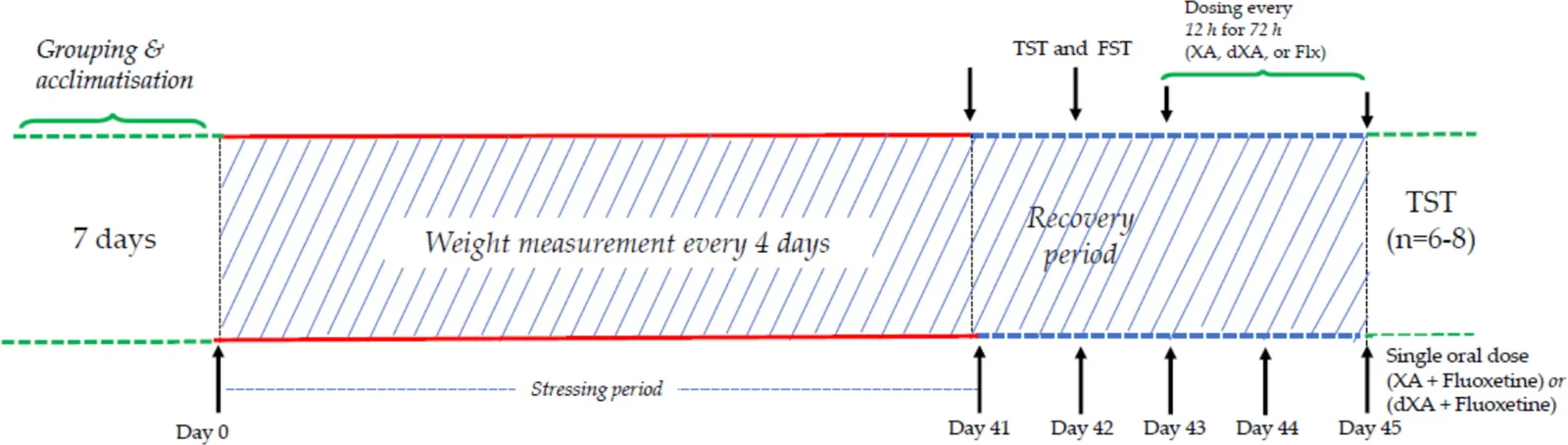
Timeline for chronic restrain stress protocol. Stressingperiod wasfrom day 0 to day 41 (42 days) with a24-h rest period before behavioural testing on day 42.To ascertain whichof the two tests ismore sensitive at detecting behavioural deficits in the experimental mice, on day 42, stress naive and stressed mice were subjected to testing in the tail suspension test and immediately after the forced swim test. The mice were then randomised into a single dosing group of multiple dosing regimen group. The randomised mice dosed in either arm were tested in the TST (the more sensitive test) 90 min after the last dose

Body weights were recorded every 4 days throughout the 42‑day stress protocol. The impact of chronic restraint stress on depressive‑like behaviour was evaluated using the forced swim test and tail suspension test as previously described. A schematic overview of the experimental timeline is presented in Fig. 2.

### Weight change

The weight of all experimental animals was taken at 0700 h on the first day and every four (4) days for the 42-day experimental period. The changes in weight were recorded to the nearest 0.1 g.

### Behavioural tests

On day 42, both stress‑naïve and chronically stressed mice (*n* = 10) were randomly selected and assessed in the tail suspension test and forced swim test following the methods of Benneh et al. (2024). Behaviour of mice was recorded for 6 min, and immobility time was quantified during the final 4 min of each session. This was to assess the sensitivity of both tests in registering the behavioural deficit, if any, after the stressing period. Sensitivity, in this context, refers to the ability of immobility time in each test to accurately reflect both the presence and magnitude of stress, thereby providing optimal discrimination between stressed and stress-naïve mice.

After testing in the FST and TST, chronic restraint stress, mice exhibited a general trend towards decreased mobility. This effect was statistically significant relative to stress-naïve controls in the tail suspension test, but not in the forced swim test; therefore, subsequent assessments were carried out in the forced swim test.

Following behavioural testing on day 42, mice were regrouped into two groups—group A for single compound testing and group B for combination compound testing. Stressed mice in group A (*n* = 6–7) received an oral administration of fluoxetine (30 mg kg^−1^) every 12 h for 72 h. Similarly, stressed mice in group A received either XA (100 mg kg^−1^), dXA (100 mg kg^−1^) or solvent (10 mL kg^−1^) every 8 h for 72 h prior to subsequent behavioural evaluation in the tail suspension test.

On day 45, an additional cohort of stressed mice, group B (*n* = 7), received a single combination dose consisting of fluoxetine (30 mg kg^−1^) with either XA (100 mg kg^−1^) or dXA (100 mg kg^−1^), while stress‑naïve mice were receiving solvent (10 mL kg^−1^, *p.o*.) 90 min before behavioural testing.

Ninety minutes after the final oral dose in either group A or, mice were suspended by the tail (1 cm from the tip) using adhesive tape affixed to a horizontal bar positioned 50 cm above the surface. Behaviour was recorded for 6 min, and immobility time was analysed during the last 4 min of each session.

## Statistics and data analysis

All data are represented as mean ± SEM, and differences between groups were analysed using a one-way analysis of variance (ANOVA) with a Sidak post hoc test unless stated otherwise. The antidepressant-like activity for each treatment was calculated as a percentage reduction in immobility.

Each point in the dose–response curves represents the mean ± SEM of the percentage reduction in immobility (*n* = 6).

The potencies (ED_50_) of the investigational drugs were determined from dose–response curves fitted using an iterative nonlinear regression (3-parameter logistic) equation:

$$Y=a+ \frac{b-a}{1+{10}^{log{ED}_{50}-x}}$$

where:

- *a* = *Y* value at the bottom plateau
- *b* = *Y* value at the top plateau
- ED_50_ = *X* value when response is halfway between *a* and *b*

Experimentally determined ED_50_ values for the co-administered compounds (ED_50mix_) were determined from dose–response curves fitted similarly as described above.

Results used in the isobolograms were analysed according to methods described by Tallarida (2006). The ED_50mix_ and ED_50add_ values were compared using a Student’s *t-*test for independent means. Isobolograph calculations were performed in Microsoft® Excel® and subsequently plotted using GraphPad Prism for Windows version 6 (GraphPad Software, San Diego, CA, USA). For all comparisons, a *P-*value < 0.05 was considered significant.

## Results

### Combination study

#### Dose–response in the tail suspension test and forced swim test

Pre-treatment with dXA and selected antidepressants reduced immobility duration compared to solvent-treated control in both tests.

**Fig. 3:**
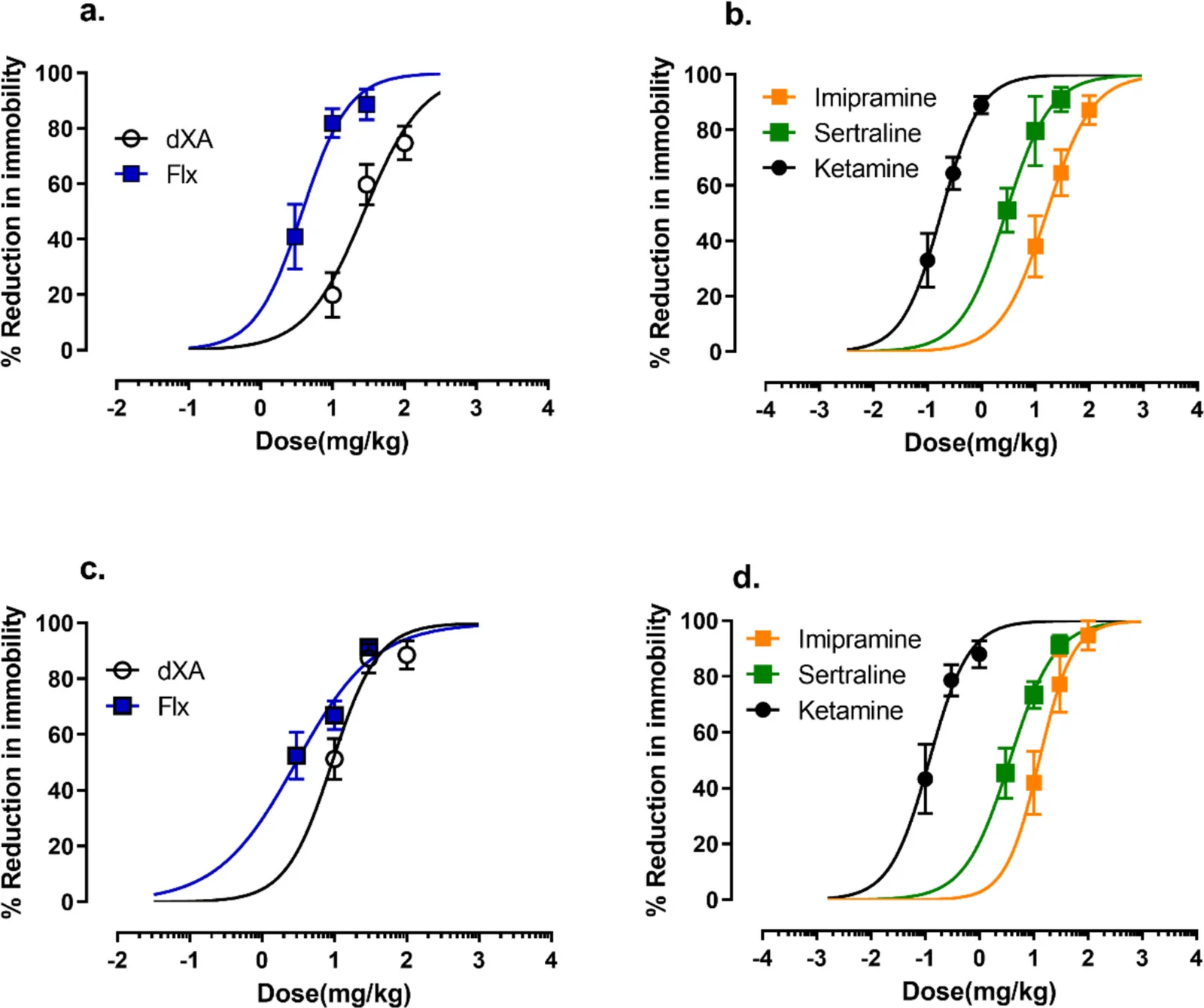
Effect of oral treatment of **a**, **c** dXA (10–100 mg kg^−1^), fluoxetine (3–30 mg kg^−1^), **b**, **d** imipramine (10–100 mg kg^−1^), sertraline (3–30 mg kg^−1^) and ketamine (0.1–1 mg kg.^−1^) on the percentage reduction in immobility in the tail suspension test (upper panel) and forced swim test (lower panel)

Ketamine was the most potent whilst the dXA was the least potent, second to imipramine, at reducing the duration of immobility in the tail suspension test. The order of potency based on the ED_50_ from the log dose–response curves is Ket < Sert < Flx < Imi < dXA (Fig. 3a and b). A similar order of potency was observed in the forced swim test: Ket < Flx < Sert < dXA < Imi, except for imipramine being less potent than dXA (Fig. 3c and d).

#### Combination dose–response in the TST and FST

**Fig. 4:**
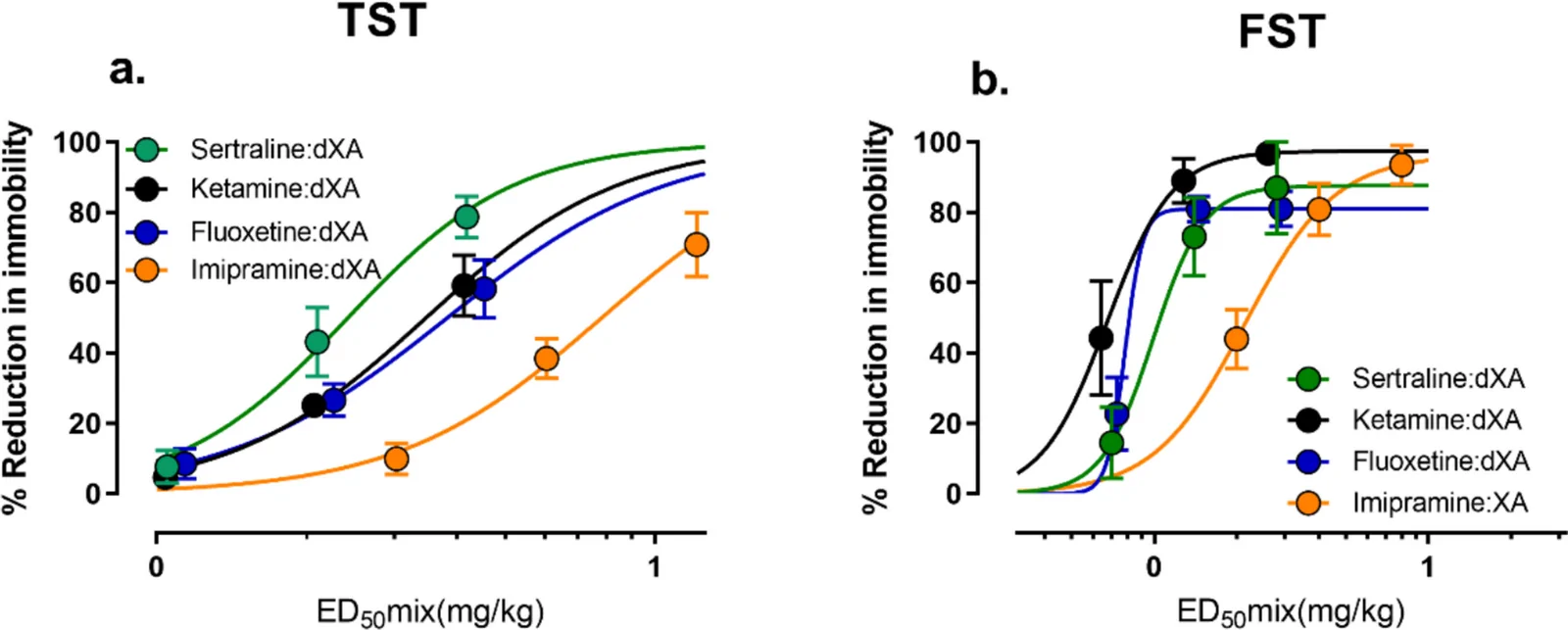
Dose–response curves for the antidepressant-like activity induced by co-administration of xylopic acid or dXA with sertraline, ketamine, fluoxetine or imipramine in the **a** tail suspension test and **b** forced swim test

The dose combinations used decreased the duration of immobility in both tests. The percentage reduction in immobility of fixed fractional doses based on the ED_50_ values (Table 1) was used to construct dose–response curves. The dose of the mixture necessary to elicit 50% of the maximal response (ED_50mix_) was determined using iterative curve fitting. dXA exhibited superior potency relative to the other antidepressants, particularly when co-administered with sertraline in the tail suspension test and with ketamine in the forced swim test (Table 2). Enhanced efficacy was also observed when dXA was combined with fluoxetine, sertraline, ketamine or imipramine (Fig. 4a and b).

**Table 1:** ED_50_ values of xylopic acid, dXA, fluoxetine, imipramine, sertraline and ketamine in the tail suspension and forced swim tests. Values represented as ED_50_ ± SEM (*n* = 6)

Compound	TST (mg kg^−1^)	FST (mg kg^−1^)
dXA	27.70 ± 5.48	9.30 ± 1.76
Fluoxetine	3.80 ± 0.80	2.96 ± 0.86
Imipramine	16.26 ± 3.97	12.61 ± 2.80
Sertraline	2.85 ± 0.97	3.62 ± 0.73
Ketamine	0.18 ± 0.03	0.12 ± 0.03

**Table 2:** ED_50mix_ values from dose–response curves of the dXA combinations tested. Values represented as ED_50_ ± SEM (*n* = 6)

Test	dXA: fluoxetine	dXA: imipramine	dXA: sertraline	dXA: ketamine
TST	11.63 ± 0.71	14.17 ± 1.34	8.81 ± 0.78	10.63 ± 0.58
FST	3.19 ± 0.15	3.02 ± 0.33	2.75 ± 0.19	2.17 ± 0.15

#### Isobolographic analysis of combination doses the TST and FST

**Fig. 5:**
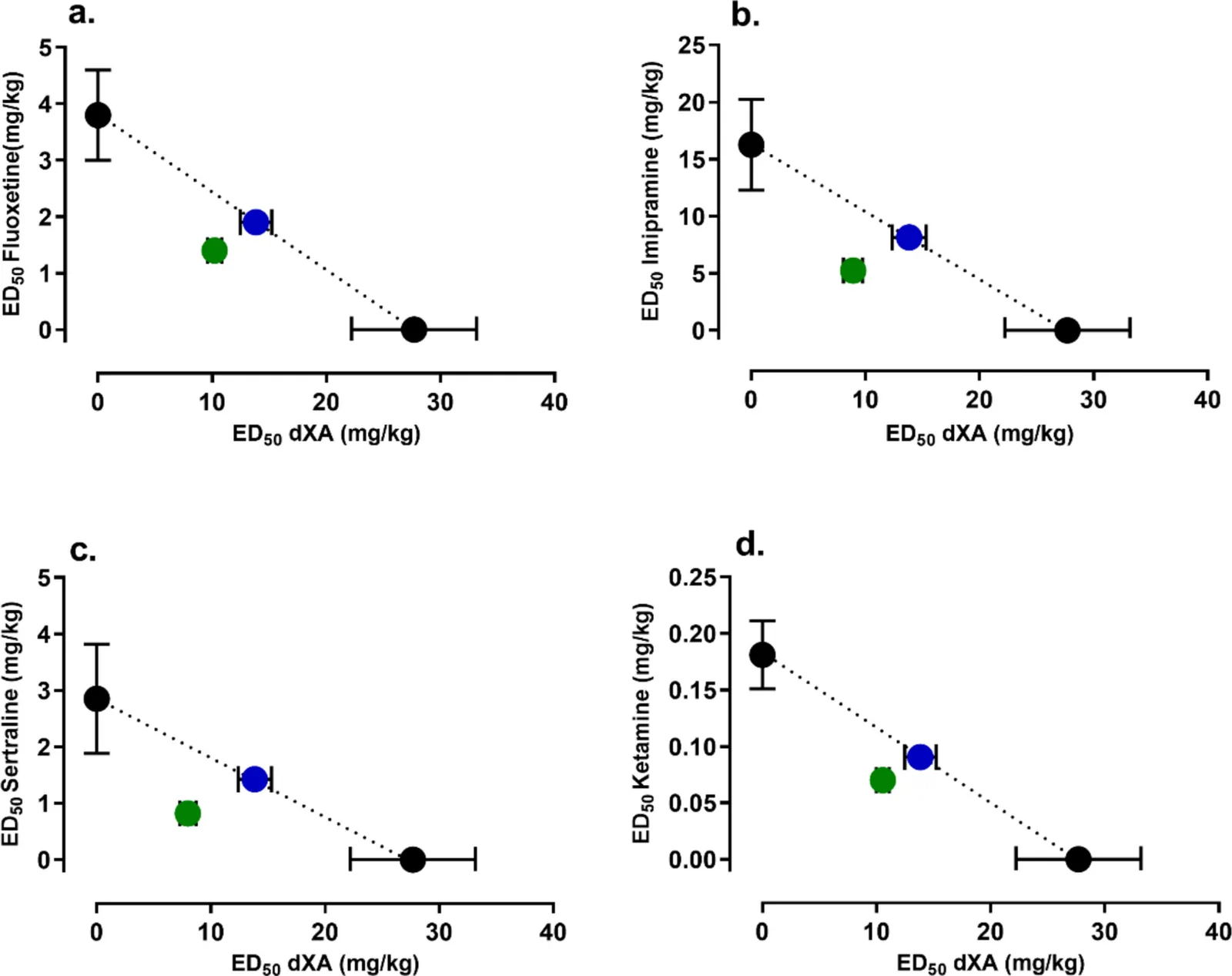
Isobolograms for the oral co-administration of dXA with antidepressants **a** fluoxetine, **b** imipramine, **c** sertraline and **d** ketamine in the tail suspension test. The theoretical ED_50_ for an additive effect (ED_50add_—*blue dot*) and the experimental ED_50_ values (ED_50mix_—*green dot)* are represented on each plot. The horizontal and vertical bars indicate the SEM

The combination of dXA with all tested antidepressants, except ketamine, revealed a synergistic effect in the tail suspension test. The synergistic interaction is demonstrated by the significant difference between the experimental ED_50mix_ and the theoretical ED_50add_ for dXA + Flx (*t*(10) = 2.39, *P* < 0.05), dXA + Imi (*t*(10) = 2.88, *P* < 0.01) and dXA + Sert (*t*(10) = 3.61, *P* < 0.01) (Fig. 5). However, the interaction of dXA with ketamine showed an additive since there was no significant difference between ED_50mix_ and ED_50add_ (*t*(10) = 2.21, *P* > 0.05).

**Fig. 6:**
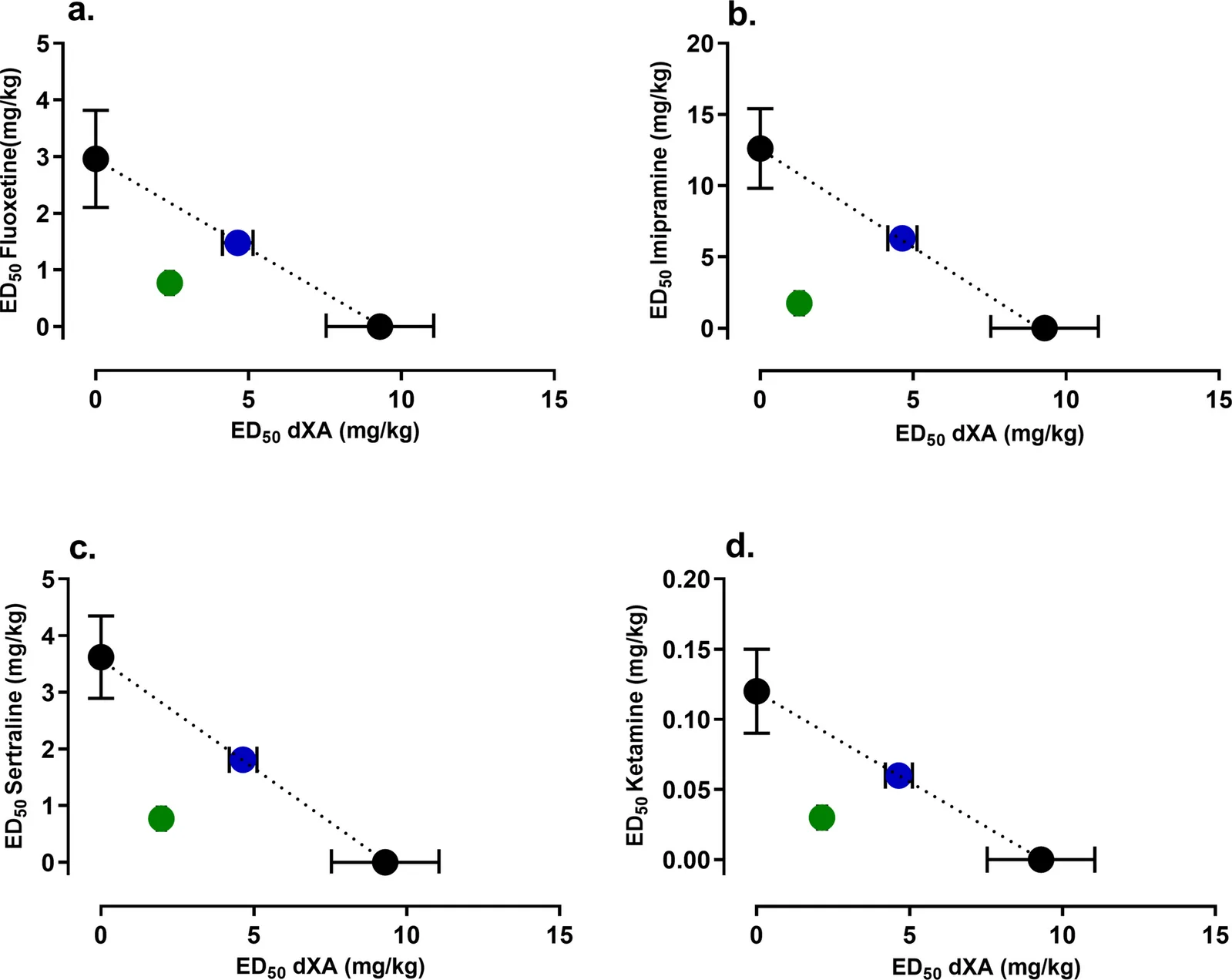
Isobolograms for the oral co-administration of xylopic acid with antidepressants **a** fluoxetine, **b** imipramine, **c** sertraline and **d** ketamine in the forced swim test. The theoretical ED_50_ for an additive effect (ED_50add_—*blue dot*) and the experimental ED_50_ values (ED_50mix_—*green dot*) are represented on each plot. The horizontal and vertical bars indicate the SEM

In a similar fashion, the combination of dXA with the selected antidepressants showed synergistic effect, in the forced swim test, when combined with fluoxetine (*t*(10) = 4.34, *P* < 0.01), imipramine (*t*(10) = 6.68, *P* < 0.001), sertraline (*t*(10) = 5.72, *P* < 0.001) and ketamine (*t*(10) = 5.35, *P* < 0.001) (Fig. 6).

The combinations (XA + Flx, XA + Imi, XA + Sert) tested in the tail suspension test did not alter the total distance traversed in the 5-min period (upper panel).

**Fig. 7:**
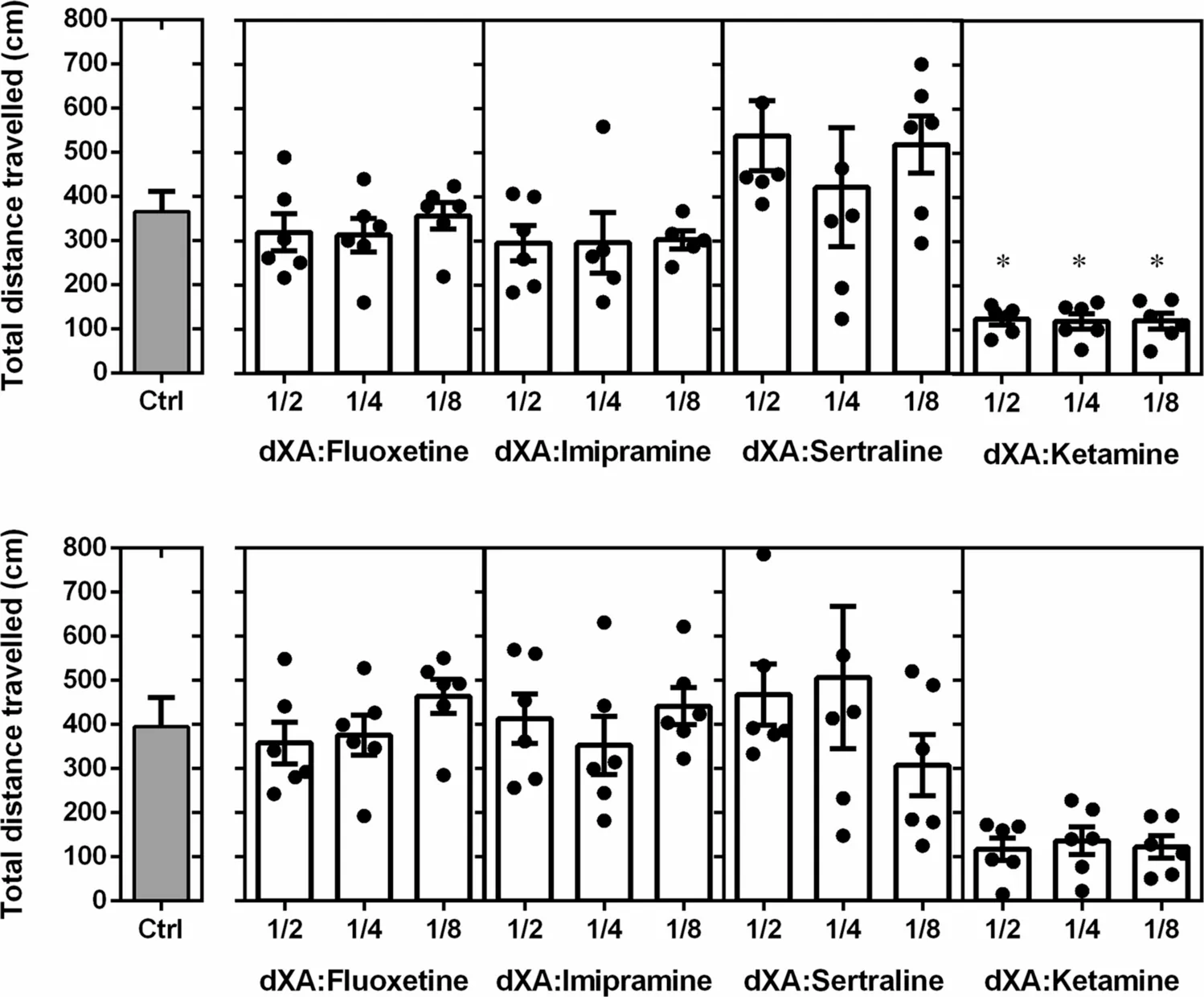
Effects of oral treatment of fixed fractional doses of dXA combinations used in the tail suspension test (upper panel) and forced swim test (lower panel) on the total distance traversed over a 5-min period in the open field test. Significantly different from control: *P < 0.05 (one-way ANOVA followed by Sidak post hoc test)

Combinations of doses of dXA with fluoxetine, imipramine and sertraline used in either the TST (Fig. 7, upper panel) or FST (Fig. 7, lower panel) did not alter the locomotor activity in the open field test. However, combination of ketamine with dXA reduced significantly the total distance traversed (Tables 3 and 4).

**Table 3:** Theoretical and experimental ED_50_ ± SEM of dXA in the tail suspension test with corresponding computed indices

Combinations	dXA/Flx	dXA/Imi	dXA/Sert	dXA/Ket
Theoretical ED_50_ (mg/kg)	15.75 ± 1.57	21.98 ± 2.36	15.27 ± 1.61	13.94 ± 1.38
Experimental ED_50_ (mg/kg)	11.63 ± 0.71	14.17 ± 1.34	8.81 ± 0.78	10.63 ± 0.58
Interaction index	0.74	0.64	0.58	0.76
Drug ratio	7.3:1	1.7:1	9.7:1	152.8:1

**Table 4:** Theoretical and experimental ED_50_ ± SEM of dXA in the forced swim test with corresponding computed indices

Combinations	dXA/Flx	dXA/Imi	dXA/Sert	dXA/Ket
Theoretical ED_50_ (mg/kg)	6.13 ± 0.66	10.95 ± 1.14	6.46 ± 0.62	4.71 ± 0.45
Experimental ED_50_ (mg/kg)	3.19 ± 0.15	3.02 ± 0.33	2.75 ± 0.19	2.17 ± 0.15
Interaction index	0.52	0.28	0.43	0.46
Drug ratio	3.1:1	0.7:1	2.6:1	77.9:1

### Chronic restrain stress model of depression

The behavioural effect of mice following chronic restrain stress was assessed at the end of the stressing period in the tail suspension test and forced swim test. A subsequent testing was conducted in the tail suspension test after either a single combination treatment with fluoxetine with either XA or dXA or a 3-day treatment with XA, dXA or fluoxetine.

**Fig. 8:**
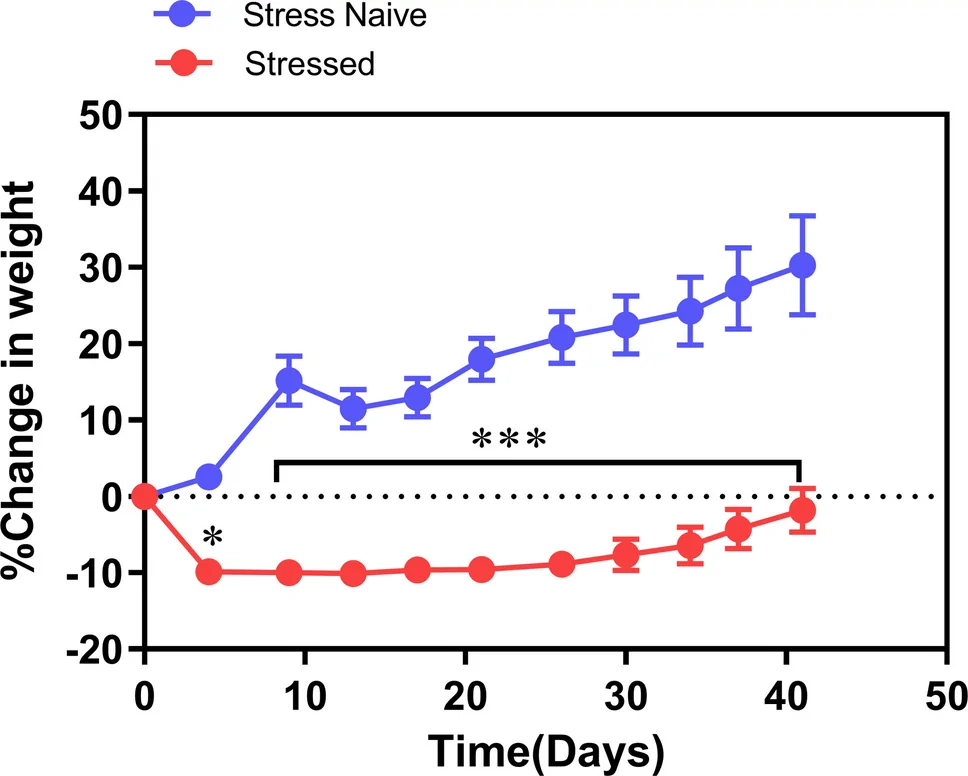
Time course plot of the percentage change in weight of stressed and stress-naïve mice during the chronic restrain stress. Significantly different from stress-naïve control at the same time point: *P < 0.05, ***P < 0.001 (two-way ANOVA followed by Holm Sidak post hoc test)

There was a general increase in weight of mice not subjected to the chronic restrain stress. The increase in weight was appreciable after day 10 and increased in a somewhat linear increase till day 42 (Fig. 8). In contrast, there was a decrease in weight which was highest on day 4 in the stressed group. This mean percentage weight loss was maintained until day 26 where there was a gradual increase towards baseline (Fig. 8).

**Fig. 9:**
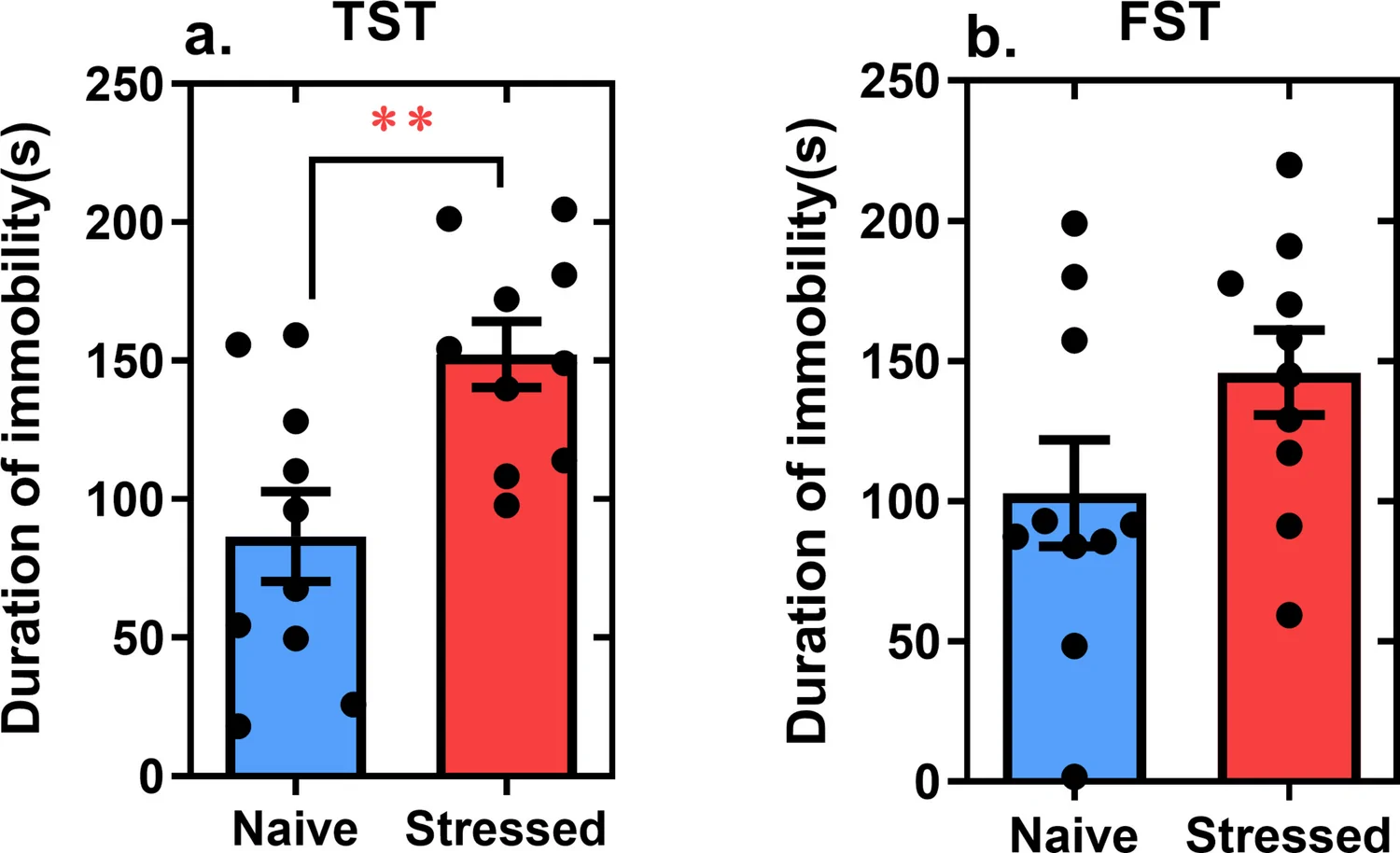
Effects of chronic restrain stress on the duration of immobility in the **a** tail suspension test and **b** forced swim test. Significantly different from naïve control: **P < 0.01 (unpaired *t*-test). Comparison showing tail suspension test was the most sensitive method for detecting depressive-like behaviour

An assessment of the behaviour of mice on day 42 of chronic restrain stress revealed a significant decrease in mobility time in of stressed mice compared to the stress-naïve control in the tail suspension test (*t*(18) = 3.27, *P* = 0.0043) but not the forced swim test (*t*(18) = 1.77, *P* = 0.094) (Fig. 9a and b). This suggests that, in this instance, the tail suspension test was the most sensitive measure for detecting depressive-like behavioural traits.

**Fig. 10:**
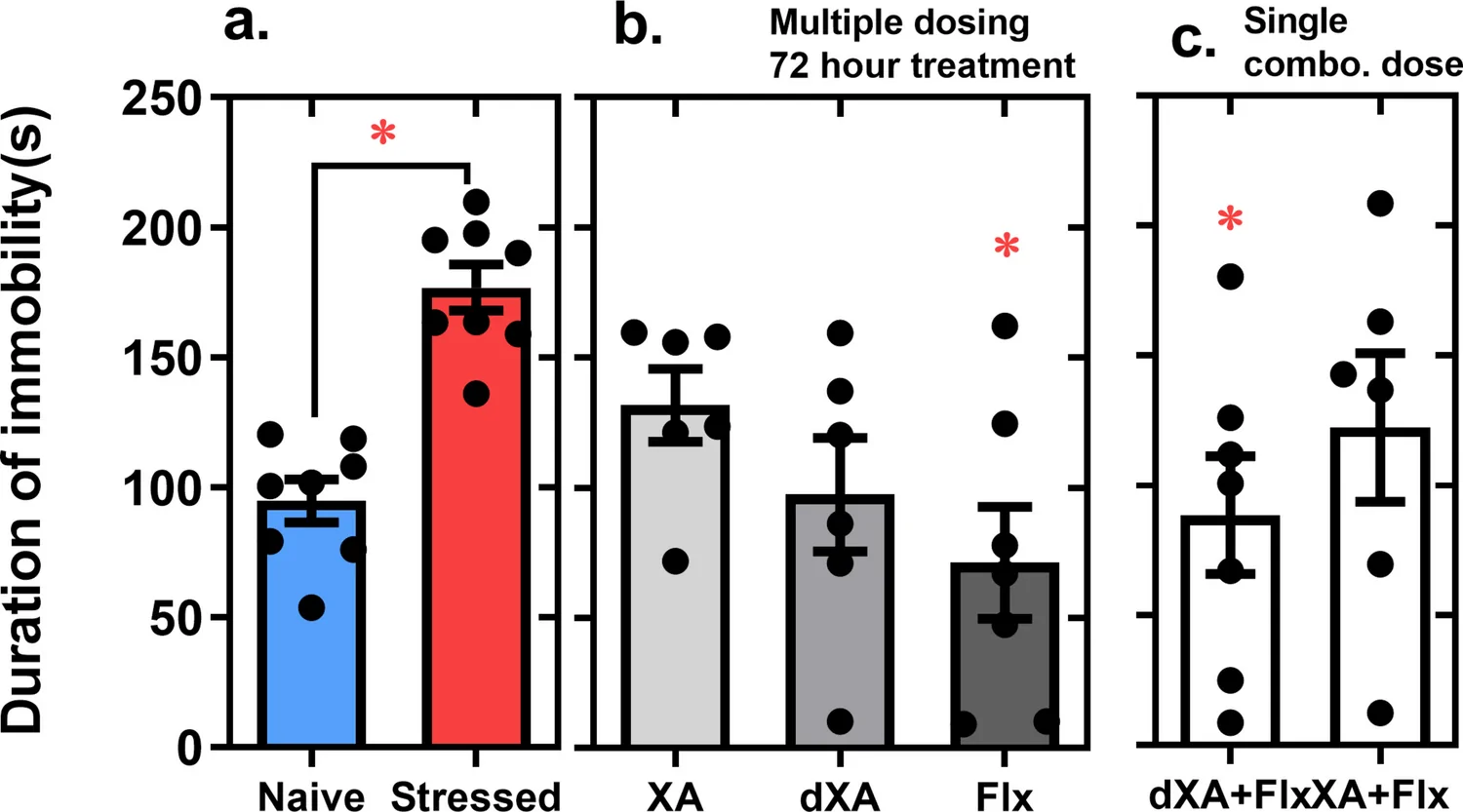
Effects of chronic restrain stress on the duration of immobility in the tail suspension test **a** before treatment and **b** after oral treatment, for 72 h, with XA (100 mg kg^−1^), dXA (100 mg kg^−1^) and Flx (30 mg kg^−1^), or **c** a single combination treatment with Flx (30 mg kg^−1^) + XA or dXA (100 mg kg^−1^). Significantly different from naïve control: *P < 0.0 (one-way ANOVA followed by Sidak post hoc test). Significantly different from stressed-control: *P < 0.05 (one-way ANOVA followed by Sidak post hoc test)

The effect of XA, dXA, fluoxetine and combinations thereof on the immobility time in the tail suspension test was assessed on day 45 (72 h post the stressing regimen). The duration of immobility in the stressed group was significantly higher (*P* < 0.05) compared to stress-naïve mice even after a 3-day non-stress period (Fig. 10a). Dosing stressed mice with 100 mg kg^−1^ of XA or dXA every 12 h for 72 h did not reverse the increase in immobility time in the tail suspension test (Fig. 10b). However, a similar treatment regimen with fluoxetine was able to reduce the immobility time significantly (*P* < 0.05).

In a similar fashion, stressed mice on day 45 were dosed with a single oral combination of fluoxetine with XA or dXA. A significant reduction in immobility was observed with the dXA + Flx (*P* < 0.05) combination but not XA + Flx (Fig. 10c).

## Discussion

The present study investigated the interaction between deacetyl xylopic acid (dXA) and selected antidepressant agents, demonstrating that combination strategies may offer enhanced therapeutic potential compared with monotherapy. Combination therapy has long been explored as a means of improving clinical outcomes in depression, particularly in cases of partial or inadequate response to single agents (Blier et al. 2010). Consistent with this rationale, our findings show that dXA, when combined with fluoxetine, sertraline or imipramine, produced antidepressant-like effects that exceeded the expected additive responses in the forced swim test (FST). Isobolographic analyses confirmed synergistic interactions for these combinations, supporting the broader concept that combination therapies can offer more rapid or effective responses than monotherapy (Carvalho et al. 2016; Cipriani et al. 2009; Blier et al. 2010).

The observed synergism between dXA and monoaminergic antidepressants (SSRIs and TCAs) likely stems from their convergent effects on neurotransmitter pathways. Previous work has established that both xylopic acid (XA) and dXA possess intrinsic antidepressant-like properties mediated primarily through serotonergic pathways (Benneh et al. 2025). This mechanistic alignment with selective serotonin reuptake inhibitors (SSRIs) such as fluoxetine and sertraline likely contribute to the observed synergism. Imipramine, which enhances both serotonergic and noradrenergic transmission, may further amplify monoaminergic signalling when combined with dXA (Lanni et al. 2009). Although the precise molecular basis of these interactions remains to be elucidated, the convergence of monoaminergic mechanisms provides a plausible explanation. Future studies incorporating neurochemical analyses, such as quantification of monoamine levels in the hippocampus, frontal cortex and hypothalamus or assessment of monoamine oxidase activity, would help clarify the mechanistic underpinnings of these synergistic effects (Duman et al. 2019).

In contrast, combinations of dXA with ketamine produced conflicting effects. Interestingly, the pattern of interaction varied between behavioural assays. While synergism was evident in the FST for dXA combined with fluoxetine, sertraline and imipramine, the tail suspension test (TST) revealed a slightly different profile, with synergistic effects observed for the same combinations but not for ketamine. While both are validated screens that use immobility as a proxy for “behavioural despair” (Can et al. 2012; Slattery and Cryan 2012), they are sensitive to different pharmacological nuances. dXA demonstrated a consistent synergistic pattern across both tests when combined with fluoxetine, imipramine and sertraline, confirming that its ability to increase escape-oriented behaviours is robust across different acute stressors (Muller et al. 2015).

These discrepancies registered for ketamine underscore the importance of employing multiple behavioural paradigms when evaluating antidepressant interactions, as each model captures distinct aspects of antidepressant-like activity (Slattery and Cryan 2012; Planchez et al. 2019). Due to the discordance in response, we classified the interaction as a potential additive effect pending further investigations.

Ketamine’s antidepressant action is mediated through glutamatergic modulation, including NMDA receptor antagonism, AMPA receptor facilitation and downstream activation of BDNF and mTOR pathways (Zhou et al. 2014; Abdallah et al. 2015). These mechanisms differ substantially from the monoaminergic pathways engaged by dXA, which may explain the absence of synergism. Moreover, ketamine’s long-term antidepressant effects typically require repeated or sustained exposure, whereas the present study employed acute dosing. The lack of synergism here provides a vital clue that the pharmacological profile of dXA likely mirrors traditional monoaminergic modulation rather than the glutamatergic plasticity-driven pathways of ketamine.

The transition from acute screening to the chronic restraint stress (CRS) model revealed a shortfall in the efficacy of XA and dXA. While fluoxetine effectively reversed CRS-induced increases in immobility, neither XA nor dXA produced significant improvements when dosed alone. This highlights a critical distinction in antidepressant research. While acute models (FST/TST) are excellent for rapid screening, testing may produce false positives for compounds with CNS stimulant activity (Pollak et al. 2010). Chronic models, which better replicate the neuroinflammatory and molecular hallmarks of human depression (Willner 2017), suggest that the current dosing regimen of dXA may not be sufficient for sustained therapeutic reversal.

This discrepancy, however, may be attributable to pharmacokinetic differences. Fluoxetine’s long half-life and active metabolite support drug accumulation and sustained exposure during repeated dosing, whereas XA and dXA have recently been reported to have rapid clearance (XA: 23–38 mL/min/kg, dXA: 19–30 mL/min/kg) and short half-lives (XA < 1 h, dXA < 2.5 h), limiting their capacity to achieve therapeutic concentrations in chronic models (Benneh et al. 2025). If their antidepressant-like effects depend on peak plasma concentration rather than sustained exposure, this would further explain their lack of efficacy in CRS (Pollak et al. 2010; Harmer et al. 2017). Consequently, the underlying mechanisms of “depression” in chronic models, which involve long-term neuroplastic changes, may require a sustained exposure that the current XA or dXA regime does not provide.

Notably, in the CRS model, dXA was able to reverse stress-induced immobility when administered as a single dose in combination with fluoxetine, using doses of both agents effective in the acute tests. This acute behavioural rescue, despite the absence of efficacy from either chronic dXA or chronic XA alone, suggests that the contribution of dXA may rely on a rapid, peak-driven psychoactive effect that becomes behaviourally meaningful only when superimposed on the neuroactive changes produced by fluoxetine exposure (Zhao et al. 2020).

An alternative explanation is that the interaction may not be purely pharmacodynamic but could instead arise from pharmacokinetic modulation. In this scenario, fluoxetine may influence the metabolism and disposition of dXA, or vice versa, through inhibition of CYP‑mediated pathways, thereby enhancing its acute behavioural effects. A similar phenomenon has been described by Fletcher and colleagues, who reported that fluoxetine, but not sertraline or citalopram, potentiated the locomotor‑stimulating effects of cocaine through CYP‑related metabolic interactions. This raises the possibility that fluoxetine or dXA may alter exposure or bioavailability in a comparable manner, contributing to the amplified behavioural response observed under acute co‑administration (Fletcher et al. 2004).

An additional consideration is that dXA may act as an active metabolite of XA, a possibility supported by their structural relationship and overlapping pharmacological profiles. If XA undergoes metabolic conversion to dXA, then the more robust acute effects observed with dXA could reflect its role as the pharmacologically dominant species. To strengthen these conclusions, future studies should incorporate pharmacokinetic–pharmacodynamic modelling to determine whether XA is converted to dXA in vivo, quantify plasma and brain levels of both compounds under acute and chronic dosing and evaluate sustained-exposure strategies such as slow-release formulations or osmotic minipumps. Challenge experiments in which dXA is administered acutely following established chronic fluoxetine treatment or CRS exposure, paired with measurements of rapid signalling events such as ERK/mTOR activation or immediate-early gene expression, would further clarify whether dXA primarily acts as a transient monoaminergic amplifier or whether it engages independent plasticity-related pathways.

## Conclusions

Taken together, these findings suggest that while XA and dXA show considerable promise in acute models and in combination with monoaminergic antidepressants, their utility in chronic depression may require pharmacokinetic optimisation or formulation strategies that enhance bioavailability and sustain exposure. The synergistic interactions observed with SSRIs and tricyclic antidepressants highlight the potential for dose-sparing combination therapies, which could improve tolerability and expand treatment options for individuals with treatment-resistant depression. Further mechanistic and pharmacokinetic studies will be essential to fully characterise the therapeutic potential of these compounds.

## Supplementary information

Below is the link to the electronic supplementary material.

ESM 1 (DOCX 297 KB)

## Data Availability

All data generated or analysed during this study are included in this published article as supplementary material.  Chromatograms and spectra for the investigational compounds have also been included in the additional file of this article.
