# Supplementary material for: Deacetyl xylopic acid exhibits synergistic antidepressant-like effects and augments fluoxetine activity in a chronic restraint-induced depression model in mice
Source: Naunyn Schmiedebergs Arch Pharmacol. 2026 Jun 5;399(11):18015–28. doi: 10.1007/s00210-026-05501-8 (PMC13503425; doi:10.1007/s00210-026-05501-8)
Supplement: Supplementary file 1 [file 210_2026_5501_MOESM1_ESM.docx]

**SUPPLEMENTARY DATA**

***A1.0 LC-MS analysis***

The spectra of xylopic acid and semi-synthetic derivatives showed a resolved peak for the compounds with ionization profiles corresponding to the accurate mass of the parent ions, daughter ions or adducts.

Figure 1: Mass determination of xylopic acid (scan mode – negative ionisation MW = 360.1 g/mol).

***A2.0 NMR analysis***

In the ^1^H NMR of the sample extracted from the fruits, xylopic acid can characteristically be confirmed by three intense methylene signals in the alkyl region as well as a hydroxyl signal downfield to signify the carboxylic acid functional group. Carbon signals on the ^13^C NMR spectrum confirms the carbon groups and functional groups similar to the hypothesised xylopic acid. The structure of the hypothesized products of synthesis were confirmed based on the data obtained from the ^1^H and ^13^C NMR spectra.

Figure 2: ^1^H spectra of xylopic acid

Figure 3: ^1^H-NMR spectrum of recrystallised compound from Xylopia aethiopica, identified as xylopic acid; with partial assignment

Figure 4: ^13^C Spectra of xylopic acid
